# Ethnic and Regional Disparities in Self-Reported Smoking Cessation Success: A Cross-Sectional Analysis of Local Authority-Level Data in England

**DOI:** 10.3390/healthcare14172715

**Published:** 2026-08-25

**Authors:** Ferozkhan Jadhakhan, Basiru Gai, Sharanya Sunil Kumar

**Affiliations:** 1 Department of Life and Sports Sciences, School of Life and Health Sciences, Birmingham City University, Birmingham B15 3TN, UK; basiru.gai@bcu.ac.uk; 2 Independent Researcher, Reading RG1 1AF, UK; drsharanya98@gmail.com

**Keywords:** smoking cessation, health status disparities, ethnic groups, England, local authority, Stop Smoking Service

## Abstract

**Highlights:**

**What are the main findings?**

- Within-category comparison. After Bonferroni correction, no statistically significant differences were observed within aggregated ethnic categories. Broad-category comparison—No statistically detectable differences were observed between the three largest aggregated ethnic categories within the precision of the available LA-level data.

**What are the implications of the main findings?**

- Need for granular ethnicity monitoring—Routine reporting should move beyond six aggregated categories to avoid obscuring communities with disproportionately low quit success and to support equitable commissioning.
- Caution in interpreting LA-level data—Broad-category absence of statistically detectable differences at Local Authority level should not be assumed to reflect individual-level equity; more detailed data are required to guide culturally responsive cessation support.

**Abstract:**

**Background/Objectives**: Smoking is the leading cause of preventable mortality in England, and NHS Stop Smoking Services (SSSs) routinely report quit outcomes using six aggregated ethnicity categories. These broad categories may obscure meaningful within-category variation. This study examined the regional distribution of English Local Authorities (LAs) and assessed ethnic disparities in self-reported quit-success at LA level. **Methods**: A cross-sectional ecological analysis was conducted using routinely published SSS data (April 2025–December 2025) for 162 English LAs. One-way ANOVA was conducted across aggregated ethnic categories. Paired-sample *t*-tests compared mean quit success between the White and Asian or Asian British categories. A Kruskal–Wallis test assessed differences across all six categories, followed by Mann–Whitney U post hoc tests with Bonferroni correction for the three comparisons performed (adjusted α = 0.017). Correlation analysis examined the relationship between quit-date setting and quit success. **Results**: London contained the largest proportion of Local Authorities (15.6%). After Bonferroni correction (α = 0.0083), none of the within-category comparisons were statistically significant. White and Asian or Asian British categories displayed highly correlated quit success rates (r = 0.708, *p* < 0.001) with no significant mean difference (t(110) = 0.99, *p* = 0.324). The Kruskal–Wallis test identified an overall difference across the six aggregated categories (H(5) = 21.67, *p* < 0.001), largely influenced by the low ranking of the Not Stated group. Quit-date setting and quit success were not meaningfully associated (r = 0.005, *p* = 0.883).

## 1. Introduction

Smoking is the single largest preventable cause of death and disease in England [1] and remains a major driver of health inequalities between social groups, ethnic groups, and geographic areas [2]. NHS Stop Smoking Services (SSSs) provide behavioural and pharmacological support to smokers attempting to quit and routinely report the number of people setting a quit date and the proportion who self-report having successfully quit at four-week follow-up, broken down by demographic characteristics including age, sex, socioeconomic classification, ethnic group, and Local Authority (LA) of residence [3].

A substantial body of evidence shows that smoking prevalence and cessation outcomes are patterned by socioeconomic position [4,5,6]. Individuals in more deprived neighbourhoods are less likely to complete treatment and less likely to achieve a successful quit through SSS support, even after adjusting for other predictors [7], although deprived groups are also more likely to be offered and to take up cessation support in both primary care and specialist services, which can partly offset lower per-attempt success rates at a population level [8,9]. Tailoring behavioural interventions for socioeconomically disadvantaged smokers does not appear to meaningfully improve quit success relative to standard support once access is achieved [10].

Less consistently examined is how cessation outcomes vary by ethnic group and by region within England. National SSS statistics typically report ethnicity using six broad, aggregated categories (White; Asian or Asian British; Black or Black British; Mixed; Other; and Not Stated). However, the use of six broad aggregated categories substantially limits the ability to detect meaningful variation in cessation outcomes between the more granular constituent groups nested within them. This loss of resolution is particularly important because aggregated categories can mask communities’ disproportionately low quit success or high smoking burden, leading to misrepresentation of service performance and potentially misdirected commissioning decisions. Mathur al [11] demonstrated that broad aggregated ethnicity categories can obscure important within-group variation in health outcomes. Furthermore, prior peer-reviewed work has demonstrated that aggregated ethnicity categories can conceal substantial heterogeneity in smoking prevalence and cessation patterns [12]. Large differences in smoking prevalence could exist between the more detailed constituent groups nested within each broad category, such that reporting only aggregated category risks masking communities with disproportionately high smoking burden [12]. Similar concerns have been raised about the granularity and completeness of ethnicity recording in routinely collected English health records more broadly [13]. Smoking and cessation outcomes also vary geographically, with regional differences in smoking prevalence and socioeconomic gradients in smoking persisting across England [14]. City-level cessation campaigns have been shown to materially increase local quit attempts [2].

Routine SSS reporting combines Local Authority aggregates with broad ethnicity categories, creating important challenges for interpretation. LA-level patterns may reflect demographic composition, service configuration, or recording practices rather than individual-level disparities, and aggregated ethnicity categories can obscure meaningful variation between constituent groups. These contextual considerations motivate the need to examine how quit-success patterns appear when using the six standard aggregated categories, while recognising that LA-level data provide ecological rather than individual-level insight.

This study therefore had the following two aims: (1) to assess variation in self-reported quit success across the six standard aggregated ethnic categories at Local Authority level and (2) to compare self-reported quit success rates between the aggregated ethnic categories using parametric and non-parametric approaches. A brief description of the regional distribution of contributing Local Authorities is retained only as contextual information about the structure of the dataset.

## 2. Materials and Methods

### 2.1. Design and Data Source

This was a cross-sectional, ecological (area-level) analysis of secondary, routinely collected data describing self-reported smoking quit success rates for English Local Authorities, disaggregated by ethnic group. The dataset “Statistics on Local Stop Smoking Services, England, April 2025 to December 2025”, published in the NHS Stop Smoking Services statistical series for England, was used in this study. The dataset reports quit-date setting and four-week self-reported quit outcomes at national, regional, and Local Authority level, including breakdowns by ethnic group [3]. For the correlation analysis, the proportion of individuals setting a quit date was defined as the number of service attendees in each LA-by-ethnic-category cell who set a quit date divided by the total number of service attendees recorded in that same LA-category cell; this proportion therefore reflects engagement among service users rather than the wider LA population or all smokers. Data were extracted from Table 2.5 (“Quit outcomes by Local Authority and ethnicity”) on 5 July 2026. For each LA-by-ethnic-category observation, the numerator (number reporting successful cessation) and denominator (number setting a quit date) were extracted directly from Table 2.5 of the NHS dataset, except where values were suppressed for disclosure control. These numerators and denominators are reported in Supplementary File S3 to allow readers to assess the precision of each percentage estimate.

It comprised all 218 LAs reported in the relevant SSS data series, distributed across nine English regions plus one national (England) summary row. The working dataset contained 162 Local Authorities with complete ethnicity-specific quit-success data. Regional and national summary rows were excluded because they do not represent individual Local Authorities. Self-reported quit-success rate was defined, consistent with SSS reporting conventions, as the percentage of those setting a quit date who self-reported having quit smoking at four-week follow-up [3]. Ethnic group was recorded using six standard aggregated categories.

### 2.2. Statistical Analysis

Descriptive statistics (counts and percentages) summarise the distribution of LAs across English regions. Six one-way ANOVA models were conducted to assess variation in mean self-reported quit success across Local Authorities within each of the six aggregated ethnic categories. The numbers of Local Authorities contributing to six category-specific analyses were White (n = 162), Asian or Asian British (n = 111), Black or Black British (n = 72), Mixed (n = 85), Other ethnic group (n = 70), and Not Stated (n = 117). Because the dataset contains only the six aggregated ethnic categories and does not provide detailed constituent ethnicity subgroups, no within-category subgroup comparisons were performed. A Bonferroni-adjusted significance threshold (α = 0.0083) was applied to account for the six tests. Degrees of freedom are not reported because each category has a different number of contributing Local Authorities. Paired-sample *t*-tests, with accompanying Pearson correlation coefficients, compared mean LA-level quit success rates between the White and Asian or Asian British categories, treating each LA as a matched pair. A Pearson correlation was also conducted to examine the relationship between the proportion of individuals setting a quit date and the percentage reporting successful cessation across all ethnic groups (N = 972 LA-by-ethnic-group observations). The NHS dataset suppresses LA-by-ethnic-group cells where the number setting a quit date is small or unstable. As a result, not all 218 LAs contribute data for all six categories. Analyses therefore use only non-suppressed cells. The correlation analysis includes all LA-category observations with both quit-date-setting and quit-success values available (N = 972). The Kruskal–Wallis test includes only LA-category observations with non-missing success-rate values (N = 617). Because quit-success percentages are more frequently suppressed than quit-date-setting counts, more LA-category cells contribute to the correlation (N = 972) than to the Kruskal–Wallis test (N = 617); this discrepancy in sample size reflects NHS suppression rules rather than any analytic inconsistency. Additionally, LA-level percentages vary in precision depending on the number of clients contributing to each cell; we applied a minimum denominator threshold of ≥20 quit-date setters and re-ran all analyses using only observations meeting this criterion. This threshold-restricted analysis was included to address potential small-cell variance and ensure that the observed patterns were not driven by low denominators. Full-sample and threshold-restricted results are reported side-by-side.

Because success-rate distributions were not assumed to meet the normality and homogeneity-of-variance assumptions required for an omnibus comparison across all six categories simultaneously, a Kruskal–Wallis test was used to compare success-rate distributions (expressed as mean ranks) across the six ethnic categories. Assumption diagnostics were conducted for all parametric tests. Levene’s tests were used to assess homogeneity of variance, and histograms and Q-Q plots were inspected to evaluate normality. Full assumption-diagnostic outputs, including Levene’s tests, Shapiro–Wilk tests, histograms, and Q-Q plots for each aggregated ethnic category and for the paired-sample difference scores, are provided in Supplementary File S4. These diagnostics were provided to demonstrate that normality and homoscedasticity assumptions were thoroughly evaluated. Additionally, these diagnostics were performed using the LA-level quit-success percentages for each aggregated ethnic category, excluding suppressed cells. Where the Kruskal–Wallis test indicated a statistically significant overall difference, pairwise Mann–Whitney U tests were conducted post hoc between the three largest categories (White, Black or Black British, and Asian or Asian British). To address multiplicity, we defined the hypothesis family for post hoc testing as the three pairwise comparisons actually performed between the three largest ethnic categories (White, Asian or Asian British, Black or Black British). A Bonferroni correction was therefore applied for three tests (adjusted α = 0.017). For the ANOVA models, which form a separate hypothesis family, we applied Bonferroni correction for the six tests conducted (adjusted α = 0.0083), and we note that the corrected *p*-values alter the interpretation of variation across Local Authorities within each aggregated ethnic category. Unadjusted *p*-values are reported alongside adjusted thresholds for transparency non-parametric methods following Hollander et al. [15]. Analyses were conducted using IBM SPSS Statistics, version 32.0. All analyses were ecological, and LA-level aggregated data should not be interpreted as individual level associations. A CONSORT flow diagram (Supplementary File S1) documents the progression from the full set of 218 Local Authorities to the analytic samples used for each test, including the impact of NHS suppression rules on LA-category availability.

### 2.3. Ethical Considerations

This analysis used only previously published, aggregate, non-identifiable Local Authority-level statistics. No individual-level or identifiable data were analysed, and no new data were collected from human participants, which made a formal ethical approval not required for this type of secondary, aggregate-level analysis of routinely published official statistics.

## 3. Results

### 3.1. Regional Distribution of Local Authorities

As contextual background for interpreting the dataset structure, the Local Authorities contributing non-suppressed ethnicity-specific quit-success data were distributed across all English regions. The dataset included 162 Local Authorities across all English regions (Table 1). London contained the highest number of LAs (n = 34; 21.0% of all LAs), followed by the North-West (n = 25; 15.4%) and South-East (n = 20; 12.3%). The East Midlands (n = 11; 6.8%) and East of England (n = 12; 7.4%) contributed the smallest proportions. This uneven distribution reflects underlying population size and local government administrative structure rather than any feature of smoking or cessation behaviour and should be borne in mind when interpreting regional patterns elsewhere in the literature.

### 3.2. Variation in Quit Success Across Local Authorities Within Aggregated Ethnic Categories (One-Way ANOVA)

To assess whether quit success varied within each aggregated ethnic category across Local Authorities, we conducted six separate one-way ANOVA models (one per category). This approach differs from an omnibus ANOVA comparing ethnic categories against each other; instead, it evaluates within-category variation across Local Authorities. Because six independent ANOVAs were run, a Bonferroni-adjusted significance threshold of α = 0.0083 was applied. After correction, none of the six ANOVA models showed statistically significant variation in mean self-reported quit success across Local Authorities within the respective aggregated ethnic categories. Effect sizes were small (η^2^ range = 0.011–0.052), indicating limited practical differences. Threshold-restricted analyses (≥20 quit-date setters) produced the same pattern of results. Assumption-diagnostic outputs are provided in Supplementary File S4. Table 2 summarises variation in self-reported quit success across Local Authorities within each aggregated ethnic category. Each row represents a separate one-way ANOVA model evaluating whether Local Authorities differed in quit success for that specific category. No model reached statistical significance after Bonferroni correction (α = 0.0083), and effect sizes were uniformly small, indicating broadly similar quit success rates across Local Authorities within each aggregated category. A separate non-parametric Kruskal–Wallis test (Section 3.4) was used to compare quit success between the six aggregated ethnic categories.

### 3.3. Paired Comparison of White and Asian or Asian British Quit Success

LAs with data for both the White and Asian or Asian British categories (n = 111 matched pairs) showed very similar mean self-reported quit success rates (51.63% and 50.80%, respectively; Table 3). Variability in success rates was higher in the Asian or Asian British category (SD = 12.57) than in the White category (SD = 8.79). The two sets of LA-level success rates were strongly positively correlated (r = 0.708, *p* < 0.001), indicating that LAs achieving higher success rates for one category also tended to achieve higher rates for the other. Because both rates are measured within the same Local Authority, this correlation likely reflects shared LA-level influences (e.g., service configuration, practitioner mix, deprivation profile, and recording practices) rather than any ethnic-group-specific process. Although a partial correlation adjusting for LA-level deprivation could further clarify this shared-context effect, the available dataset does not include a consistent deprivation measure for all LA-category combinations.

The paired-sample *t*-test showed no statistically significant difference in mean self-reported quit success between the White and Asian or Asian British categories (mean difference = 0.84 percentage points, 95% CI −0.84 to 2.51; t(110) = 0.99, *p* = 0.324; Table 4), indicating that no statistically detectable difference was observed between categories within the precision of the available LA-level data. The corresponding effect size was very small (Cohen’s d = 0.09), indicating minimal practical difference between categories. The threshold-restricted paired-sample analysis (≥20 quit-date setters) produced the same pattern of results, with no statistically significant difference between categories.

### 3.4. Omnibus Comparison Across Six Ethnic Categories (Kruskal–Wallis Test)

The Kruskal–Wallis test compared self-reported quit-success rate rankings across all six ethnic categories simultaneously (N = 617 LA-level observations; Table 5). The White category showed the highest mean rank (339.28), followed by the Black or Black British (328.09) and Asian or Asian British (322.55) categories. The Mixed (298.12) and Other (317.84) categories had mid-range rankings, while the Not Stated category had the lowest mean rank (245.09). The Kruskal–Wallis test indicated a statistically significant overall difference in success-rate distributions across the six categories, H(5) = 21.67, *p* < 0.001; the comparatively low ranking of the Not Stated category is a plausible contributor to this overall result. A sensitivity analysis excluding the Not Stated category yielded a statistically significant omnibus result (H(4) = 10.92, *p* = 0.028), indicating that the six-category finding is not solely attributable to the heterogeneous Not Stated group. The Kruskal–Wallis effect size was small (ε^2^ = 0.035), suggesting limited practical disparity across categories. Applying the ≥20 denominator threshold did not materially alter the Kruskal–Wallis findings; threshold-restricted mean ranks and test statistics are reported in Supplementary File S3.

### 3.5. Post Hoc Pairwise Comparisons (Mann–Whitney U Tests)

Post hoc pairwise Mann–Whitney U tests compared the three largest ethnic categories directly (Table 6, Table 7 and Table 8). Rank–biserial correlations were very small for all pairwise comparisons (r\rb} range = 0.01–0.05), indicating negligible practical differences between categories. None of the three pairwise comparisons was statistically significant under either the conventional criterion (α = 0.05) or Bonferroni-adjusted criterion for the three tests (α = 0.017), indicating that no statistically detectable differences were observed between categories in these pairwise comparisons. Threshold-restricted Mann–Whitney U tests yielded the same non-significant results, confirming that small denominators did not drive the pairwise comparisons. This consistency between full-sample and restricted analyses confirms that the primary distributional patterns are robust to small-cell variability.

### 3.6. Relationship Between Quit-Date Setting and Quit Success

A Pearson correlation examined the relationship between the proportion of individuals setting a quit date and the percentage reporting successful cessation across all ethnic groups (N = 972 LA-by-ethnic-group observations). The correlation was negligible and non-significant, r(970) = 0.005, *p* = 0.883, indicating no meaningful association between quit-date setting rates and self-reported quit success across the dataset. In this context, the proportion setting a quit date refers to the share of service attendees in each LA-by-ethnic-category cell that set a quit date, rather than the proportion of all smokers or the wider LA population. This suggests that local areas achieving higher rates of engagement (quit-date setting) do not systematically achieve higher success rates, and vice versa.

## 4. Discussion

This analysis of 218 English Local Authorities produced two complementary findings. First, the ANOVA tests examining ethnic category yielded small differences in mean quit success, but none of these differences were statistically significant after applying a Bonferroni correction for six tests. The results did not indicate statistically significant differences across the aggregated ethnic categories after Bonferroni correction, indicating that aggregated ethnic categories may mask area-level variation. Second, when the broad categories were compared directly, differences between the three largest categories (White, Black or Black British, and Asian or Asian British) were small, strongly correlated, and not statistically significant, even though the omnibus Kruskal–Wallis test across all six categories indicated an overall difference driven largely by the low ranking of the Not Stated group. The finding that quit-date setting rates showed no meaningful association with quit-success rates (r = 0.005, *p* = 0.883) further highlights that service engagement and service effectiveness represent distinct dimensions of performance. Although the correlation between quit-date setting and quit success was negligible, this pattern is informative. At LA level, higher engagement with services (i.e., more people setting a quit date) does not translate into higher quit success, suggesting that the factors driving quit-date uptake may differ from those influencing successful cessation. This may reflect variation in service configuration, practitioner capacity, local recording practices, or demographic composition rather than any underlying behavioural relationship between engagement and success. The finding reinforces that quit-date setting and quit success represent distinct dimensions of service performance and should not be interpreted interchangeably. The strong correlation between White and Asian or Asian British quit-success rates should be interpreted as a shared-method or common-cause structure, reflecting LA-level contextual factors rather than implying any substantive similarity between ethnic categories. These results should be interpreted alongside existing evidence that aggregated ethnicity categories may obscure differences between more detailed constituent groups. Although the present ecological analysis did not identify statistically significant within-category variation in quit success, prior work has shown that smoking prevalence and cessation patterns can vary substantially between detailed ethnic categories [12]. Importantly, the inclusion of raw numerators and denominators (Supplementary File S3), comprehensive assumption-diagnostic outputs (Supplementary File S4), and effect sizes for all parametric and non-parametric tests, together with threshold-restricted analyses using ≥20 quit-date setters, confirms that these findings are robust and not an artefact of small-cell variability.

The present findings therefore reinforce the argument that aggregated ethnicity categories are insufficient for monitoring equity in cessation outcomes and may inadvertently obscure groups experiencing disproportionate smoking-related harm. A six-category classification may suggest broad equivalence in outcomes between, for example, the White and Asian or Asian British categories as a whole, while concealing the fact that specific constituent communities within either category experience systematically different cessation outcomes. This pattern echoes concerns raised about the granularity of ethnicity recording in English health records generally [13] and about aggregated reporting of smoking prevalence specifically [12].

The comparatively poor ranking of the Not Stated category is also noteworthy. Because this category is, by definition, heterogeneous and is likely to combine LAs and individuals with very different reasons for non-disclosure, it is difficult to draw substantive conclusions about any single population from this result. It may, however, be a useful flag for local data-quality improvement, since higher rates of missing ethnicity data have elsewhere been linked to weaker monitoring of ethnic health inequalities. Improving completeness of ethnicity recording may therefore be a necessary precursor to improving equity in cessation outcomes.

The absence of statistically detectable differences between the White, Black or Black British, and Asian or Asian British categories exists. These non-significant results do not imply equivalence between categories; formal equivalence testing (e.g., TOST) was not conducted. This pattern aligns with prior evidence suggesting that socioeconomic position may be a stronger driver of within-service inequalities than broad ethnic category [9,16]. It does not, however, imply that ethnic inequalities in smoking-related harm are absent: prior work shows that smoking prevalence itself varies considerably by detailed ethnic categories [12], that neighbourhood deprivation is associated with reduced treatment completion and reduced quit success [7], and that the populations reached by services, rather than only their per-attempt success rates, must be considered when assessing equity [9,16]. This highlights the importance of distinguishing inequalities in service reach from inequalities in service effectiveness, both of which contribute to population-level smoking disparities.

The regional distribution of LAs presented here provides useful context for interpreting national and regional SSS statistics as follows: because London and the North-West alone account for over a quarter of all English LAs, regional patterns in cessation activity and outcomes can be disproportionately influenced by these areas. This is consistent with prior evidence of substantial regional variation in smoking-related socioeconomic gradients across England [14] and with evidence that city-wide cessation campaigns can materially shift local quit attempt rates [2]. Understanding these regional patterns is essential for interpreting national statistics, which may otherwise obscure local variation in service performance and population need.

From a practice perspective, these findings support two complementary recommendations. First, local and national reporting of smoking cessation outcomes by ethnicity should, wherever sample size allows, move beyond the standard six-category classification to examine detailed constituent ethnic categories. Second, given the apparent absence of statistically detectable differences in outcomes between the three largest aggregated categories once directly compared, commissioners should be cautious about assuming that broad-category non-significant differences necessarily indicate the absence of disparities at a more granular level, particularly given prior evidence of ethnic category variation in smoking prevalence and cessation patterns. Routine use of more detailed ethnicity categories would allow commissioners to identify specific communities with disproportionately low quit success and target culturally tailored interventions more effectively. Across all parametric and non-parametric tests, effect sizes were uniformly small, reinforcing that observed statistical differences were of limited practical magnitude.

## 5. Limitations

This study has several important limitations. First, and most fundamentally, the unit of analysis was the Local Authority rather than the individual smoker. Associations and differences observed at this aggregate, ecological level cannot be assumed to hold at the individual level, and inferring individual-level disparities directly from LA-level comparisons risks the ecological fallacy [17]. An LA-level finding of no significant difference between two broad ethnic categories does not establish that individuals from those categories experience equivalent treatment, access, or outcomes within services [18]. Future work using individual-level data is therefore essential to validate whether the area-level patterns observed here reflect genuine disparities in quitting behaviour.

Second, the outcome measure was a self-reported four-week quit status rather than a biochemically validated (e.g., carbon-monoxide-verified) outcome. Self-report can overestimate true abstinence and may differ systematically between groups in ways unrelated to true quitting behaviour. This limitation may be particularly relevant when comparing across diverse ethnic groups if cultural or social norms influence self-reporting.

Third, although the dataset used in this study is fully documented and reproducible, the use of aggregated ethnic categories limits the ability to examine finer-grained heterogeneity within groups. Future work using individual-level data with detailed ethnicity coding would allow more precise assessment of within-category variation.

Fourth, although a Bonferroni correction was applied to the post hoc pairwise comparisons, this approach is conservative and does not eliminate the broader risk of Type I error across the full set of analyses reported, several of which were not pre-registered as a single coordinated hypothesis set. A pre-registered analysis plan would strengthen future work.

Fifth, this was a cross-sectional analysis and cannot speak to trends over time; given evidence of recent shifts in regional and socioeconomic smoking inequalities [14], repeating this analysis with multiple years of data would strengthen confidence in the stability of the patterns observed.

Sixth, LAs with small minority populations are more likely to have suppressed data, which may bias comparisons.

Finally, the Not Stated category is inherently heterogeneous, and findings relating to it should be interpreted as a signal for data-quality investigation rather than as a substantive ethnic disparity finding. The sensitivity analysis excluding Not Stated produced a statistically significant omnibus result, indicating that the observed differences between categories are not solely driven by the heterogeneous Not Stated group.

## 6. Conclusions

Across 218 English Local Authorities, the one-way ANOVA did not identify statistically significant differences across the aggregated ethnic categories after Bonferroni correction. When the three broad aggregated categories were compared directly, no statistically detectable differences were observed within the precision of the available LA-level data, although these non-significant results do not imply equivalence between categories. The absence of any meaningful relationship between quit-date setting rates and quit-success rates further underlines the importance of examining service effectiveness and service reach as distinct dimensions. These findings reinforce the importance of disaggregated ethnicity monitoring in smoking cessation services and add to growing evidence that standard aggregated ethnicity categories can mask meaningful health-related heterogeneity in England. They also highlight the need for commissioners to interpret non-significant differences between aggregated categories with caution, as similar LA-level percentages may conceal substantial disparities within constituent communities. Future research using individual-level, biochemically validated outcome data, explicit and transparent ethnic category definitions, and longitudinal designs are needed to confirm whether these area-level patterns reflect genuine disparities in access, quality of support, or quitting behaviour at the individual level. Such work would provide a stronger evidence base for equitable commissioning and culturally responsive cessation support. For policymakers, these findings emphasise the need to strengthen the granularity and completeness of ethnicity recording within Stop Smoking Services, as reliance on broad aggregated categories may obscure important area-level variation. Local Authorities could improve equity by routinely reviewing detailed ethnicity breakdowns when commissioning services and prioritising targeted support in areas where quit success is consistently lower. Reducing “Not Stated” ethnicity reporting and improving data quality would also enhance the accuracy of performance monitoring and support more responsive, culturally appropriate cessation provision.

## Figures and Tables

**Table 1:** Regional distribution of Local Authorities in England.

Region	Number of LAs	Percent of All LAs
East Midlands	11	6.8%
East of England	12	7.4%
London	34	21%
North-East	13	8.0%
North-West	25	15.4%
South-East	20	12.3%
South-West	16	9.9%
West Midlands	15	9.3%
Yorkshire and Humber	16	9.9%
Total	162	100%

Abbreviation: LA = Local Authority.

**Table 2:** Variation in self-reported quit success rates across Local Authorities within aggregated ethnic categories (one-way ANOVA).

Broad Ethnic Category	Mean	SD (+/−)	N	F Statistic	*p* Value	Interpretation
White	52.5	9.2	162	4.08	0.019	Not significant after Bonferroni correction
Asian or Asian British	50.8	12.6	111	3.40	0.036	Not significant after Bonferroni correction
Black or Black British	51.6	11.6	72	3.10	0.048	Not significant after Bonferroni correction
Mixed	50.0	11.6	85	1.64	0.198	Not significant
Other Ethnic Group	50.9	14.4	70	0.84	0.434	Not significant
Not Stated	46.1	14.7	117	0.80	0.451	Not significant

Note: Bonferroni-adjusted threshold α = 0.0083.

**Table 3:** Descriptive statistics and correlation for paired ethnic-category quit-success rates.

Measure	White (%)	Asian or Asian British (%)
Mean	51.63	50.80
N	111	111
Standard deviation	8.79	12.57
Standard error	0.83	1.19

Note. Paired-sample correlation: r = 0.708, *p* < 0.001.

**Table 4:** Paired-sample test comparing quit success between White and Asian or Asian British categories.

Comparison	Mean Diff. (%)	95% CI	t	df	*p* (2-Tailed)
White—Asian/Asian British	0.84	−0.84 to 2.51	0.99	110	0.324

**Table 5:** Success-rate rankings across ethnic categories (Kruskal–Wallis test).

Ethnic Category	N	Mean Rank
White	162	339.28
Asian or Asian British	111	322.55
Black or Black British	72	328.09
Mixed	85	298.12
Other	70	317.84
Not Stated	117	245.09
Total	617	-

Note: H(5) = 21.67, *p* < 0.001.

**Table 6:** White vs. Black or Black British (Mann–Whitney U test).

Category	N	Mean Rank	Sum of Ranks
White	162	118.69	19,228.00
Black or Black British	72	114.82	8267.00
Total	234	-	-

Note. U = 5639, Z = −0.40, *p* = 0.686.

**Table 7:** White vs. Asian or Asian British (Mann–Whitney U test).

Category	N	Mean Rank	Sum of Ranks
White	162	139.49	22,598.00
Asian or Asian British	111	133.36	14,803.00
Total	273	-	-

Note. U = 8587, Z = −0.63, *p* = 0.528.

**Table 8:** Asian or Asian British vs. Black or Black British (Mann–Whitney U test).

Category	N	Mean Rank	Sum of Ranks
Asian or Asian British	111	91.55	10,162.00
Black or Black British	72	92.69	6674.00
Total	183	-	-

Note. U = 3946, Z = −0.14, *p* = 0.886.

## Data Availability

The data are freely available via the NHS digital website: https://digital.nhs.uk/data-and-information/publications/statistical/statistics-on-nhs-stop-smoking-services-in-england/april-2025-to-december-2025-q3, last accessed on 5 August 2026.

## Supplementary Materials

The following supporting information can be downloaded at https://www.mdpi.com/article/10.3390/healthcare14172715/s1, Supplementary File S1. CONSORT Style Flow Diagram for Analytic Samples. Supplementary File S2. Summary statistics for aggregated ethnic categories used in ANOVA analyses. Supplementary File S3. Numerators and Denominators. Supplementary File S4: Assumption diagnostics for parametric tests.

## Abbreviations

The following abbreviations are used in this manuscript:

LA	Local Authority
SSSs	Stop Smoking Services
NHS	National Health Service
ANOVA	Analysis of Variance
SD	Standard Deviation
CI	Confidence Interval
dfs	Degrees of Freedom
r	Pearson Correlation Coefficient
H	Kruskal–Wallis Test Statistic
U	Mann–Whitney U Statistic
