# Supplementary material for: Ethnic and Regional Disparities in Self-Reported Smoking Cessation Success: A Cross-Sectional Analysis of Local Authority-Level Data in England
Source: Healthcare (Basel). 2026 Aug 25;14(17):2715. doi: 10.3390/healthcare14172715 (PMC13565527; doi:10.3390/healthcare14172715)
Supplement: Supplementary file 1 [file healthcare-14-02715-s001.zip › Supplementary File S4_Additional ANOVA Outputs.pdf]

Supplementary File S4: Assumption diagnostics for parametric tests

Levene's test for each ethic category

White

Tests of Homogeneity of Variances					
		Levene Statistic	df1	df2	Sig.[a]
White Percentage successful (self-reported)	Based on Mean	3.847	8	152	<.001
	Based on Median	2.988	8	152	.004
	Based on Median and with adjusted df	2.988	8	105.446	.005
	Based on trimmed mean	3.704	8	152	<.001
a. Confidence Interval: 0%					

Asian or Asian British

Tests of Homogeneity of Variances					
		Levene Statistic	df1	df2	Sig.[a]
Asian or Asian British Percentage successful (self-reported)	Based on Mean	2.062	8	101	.047
	Based on Median	1.590	8	101	.137
	Based on Median and with adjusted df	1.590	8	76.828	.142
	Based on trimmed mean	1.921	8	101	.065
a. Confidence Interval: 0%					

Black of Black British

Tests of Homogeneity of Variances					
		Levene Statistic	df1	df2	Sig.[a]
Black or Black Brtish Percentage successful (self-reported)	Based on Mean	1.282	7	62	.274
	Based on Median	.776	7	62	.610
	Based on Median and with adjusted df	.776	7	38.991	.611
	Based on trimmed mean	1.183	7	62	.325
a. Confidence Interval: 0%					

Mixed

Tests of Homogeneity of Variances					
		Levene Statistic	df1	df2	Sig.[a]
Mixed Percentage successful (self-reported)	Based on Mean	.997	7	75	.441
	Based on Median	.639	7	75	.722
	Based on Median and with adjusted df	.639	7	44.347	.721
	Based on trimmed mean	.946	7	75	.476

a. Confidence Interval: 0%

Other ethnic group

Tests of Homogeneity of Variances					
		Levene Statistic	df1	df2	Sig.[a]
Other ethnic group Percentage successful (self-reported)	Based on Mean	2.022	8	60	.059
	Based on Median	1.902	8	60	.076
	Based on Median and with adjusted df	1.902	8	38.300	.088
	Based on trimmed mean	2.015	8	60	.060

a. Confidence Interval: 0%

Not stated

Tests of Homogeneity of Variances					
		Levene Statistic	df1	df2	Sig.[a]
Not stated Percentage successful (self-reported)	Based on Mean	.968	8	107	.465
	Based on Median	.812	8	107	.594
	Based on Median and with adjusted df	.812	8	80.607	.594
	Based on trimmed mean	.933	8	107	.493

a. Confidence Interval: 0%

Histograms and Q-Q plots for each category

White

Shapiro – Wilk test

Tests of Normality						
	Kolmogorov-Smirnov[a]			Shapiro-Wilk		
	Statistic	df	Sig.	Statistic	df	Sig.
White Percentage successful (self-reported)	.050	162	.200*	.985	162	.078
a. Lilliefors Significance Correction						
*. This is a lower bound of the true significance.						

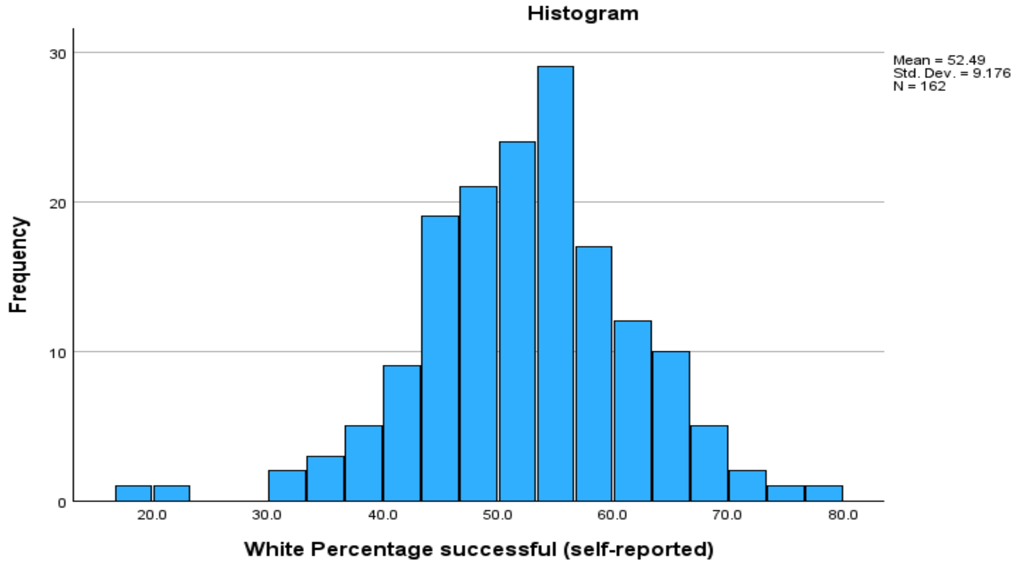

Q-Q Plot

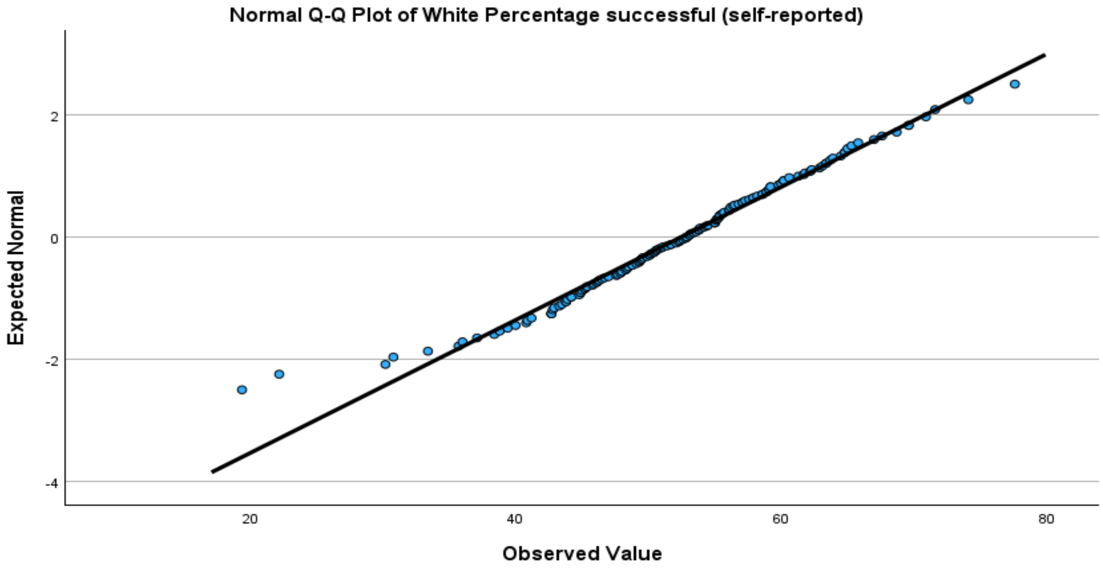

Asian or Asian British

Tests of Normality						
	Kolmogorov-Smirnov[a]			Shapiro-Wilk		
	Statistic	df	Sig.	Statistic	df	Sig.
Asian or Asian British Percentage successful (self-reported)	.111	111	.002	.959	111	.002

a. Lilliefors Significance Correction

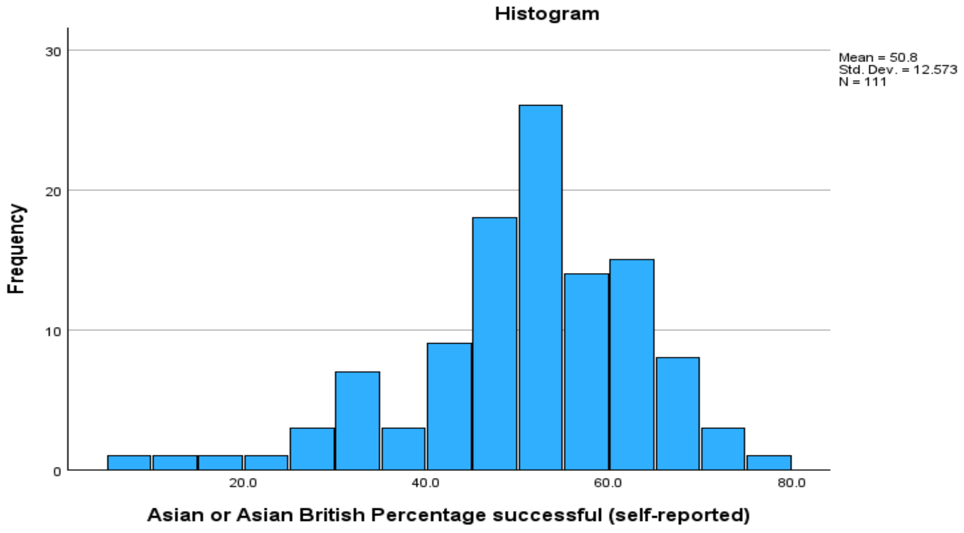

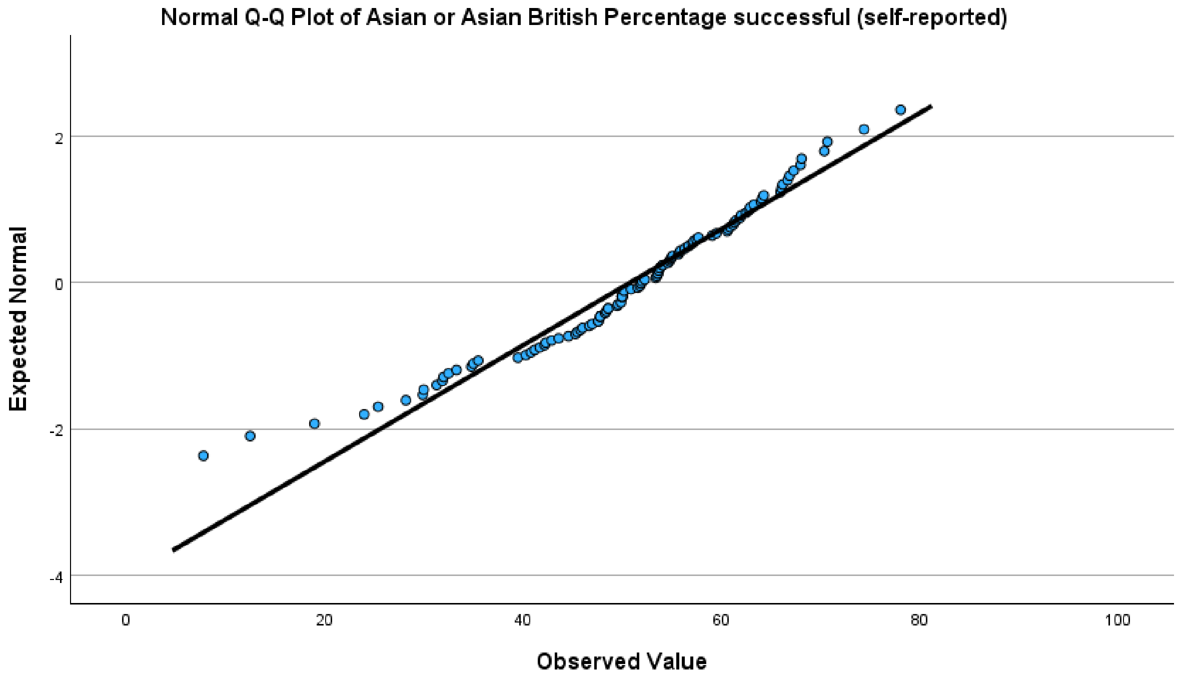

Black of Black British

Tests of Normality						
	Kolmogorov-Smirnov[a]			Shapiro-Wilk		
	Statistic	df	Sig.	Statistic	df	Sig.
Black or Black Brtish Percentage successful (self-reported)	.115	72	.019	.972	72	.115
a. Lilliefors Significance Correction						

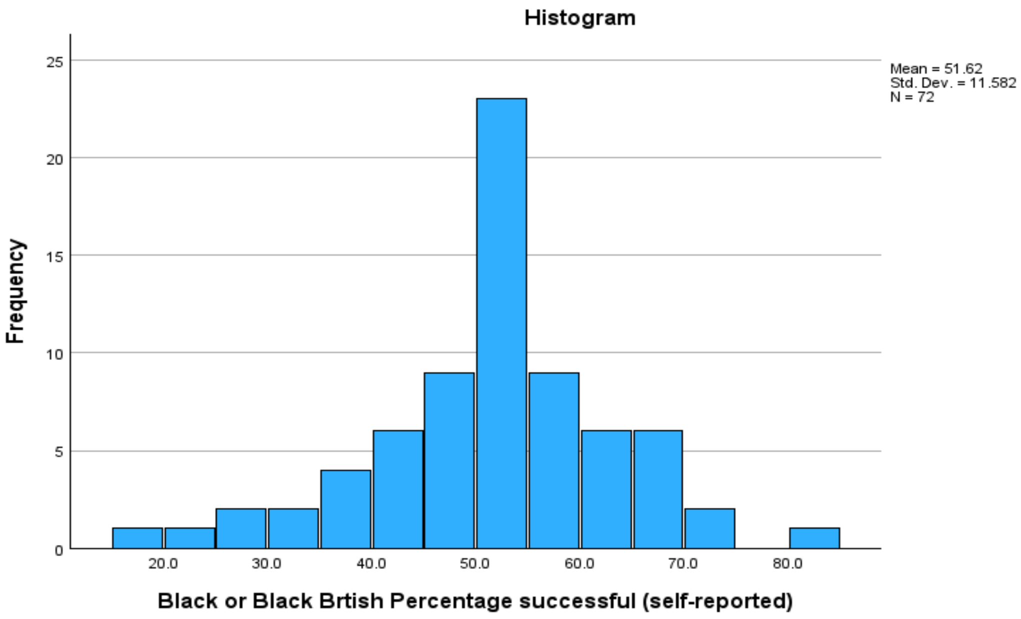

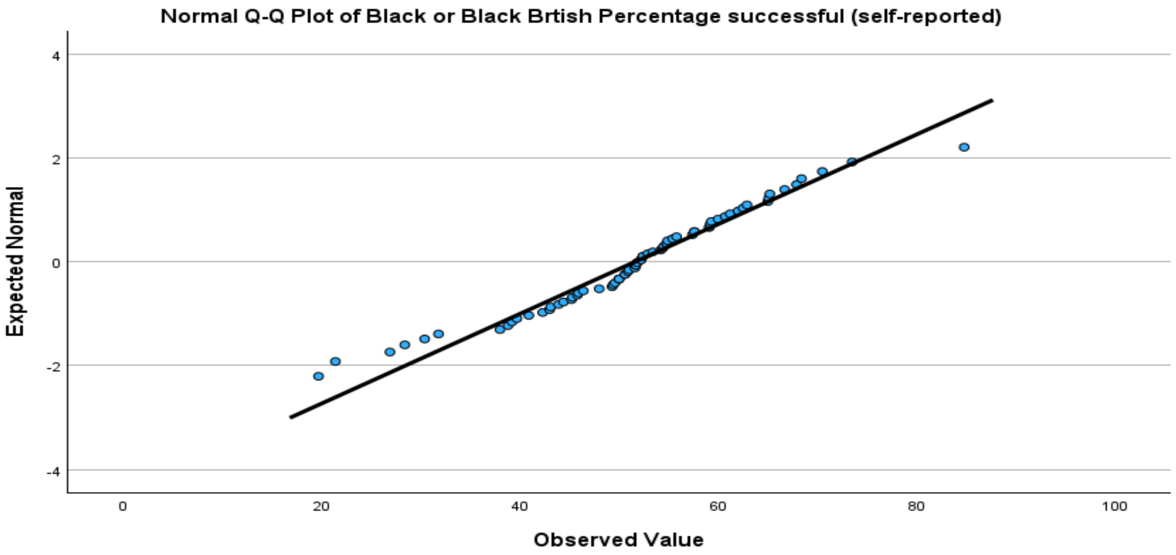

Mixed

Tests of Normality

	Kolmogorov-Smirnov[a]			Shapiro-Wilk		
	Statistic	df	Sig.	Statistic	df	Sig.
Mixed Percentage successful (self-reported)	.079	85	.200*	.983	85	.329

a. Lilliefors Significance Correction

*. This is a lower bound of the true significance.

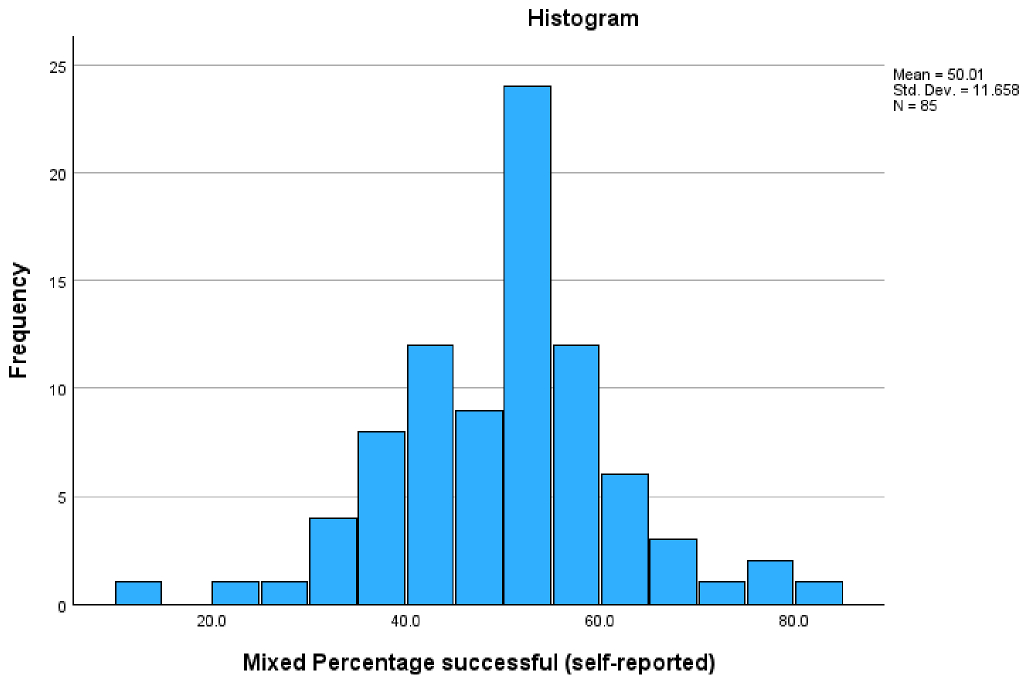

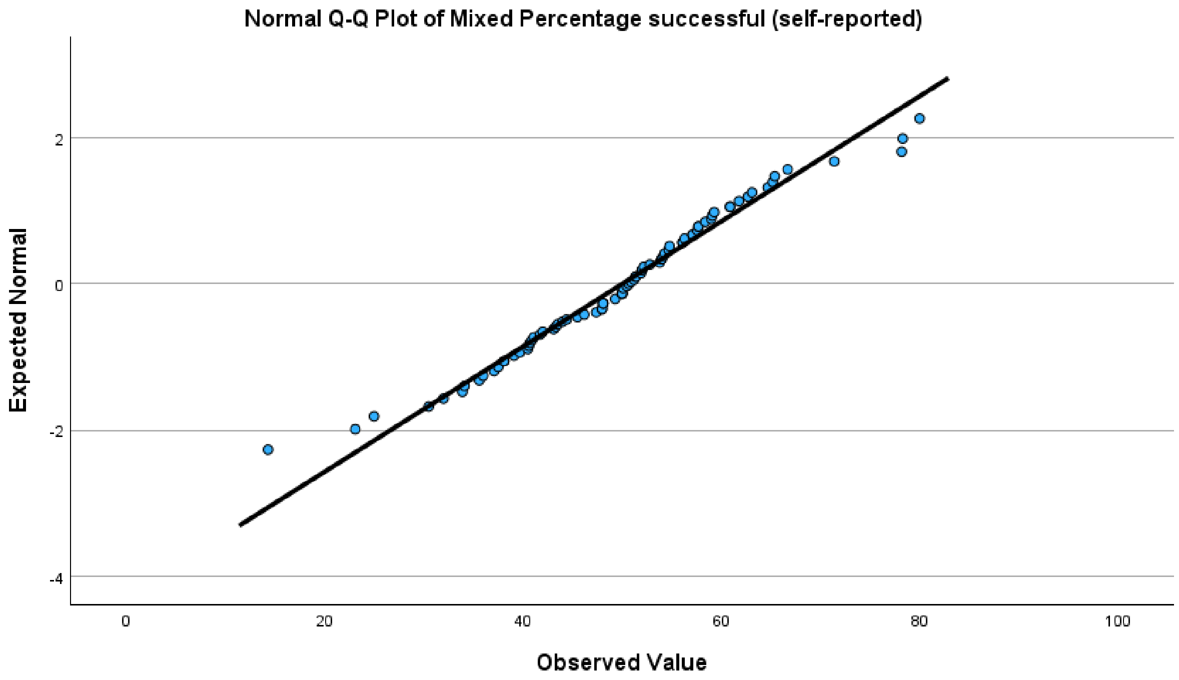

Other ethnic group

Tests of Normality						
	Kolmogorov-Smirnov[a]			Shapiro-Wilk		
	Statistic	df	Sig.	Statistic	df	Sig.
Other ethnic group Percentage successful (self-reported)	.110	70	.035	.975	70	.166

a. Lilliefors Significance Correction

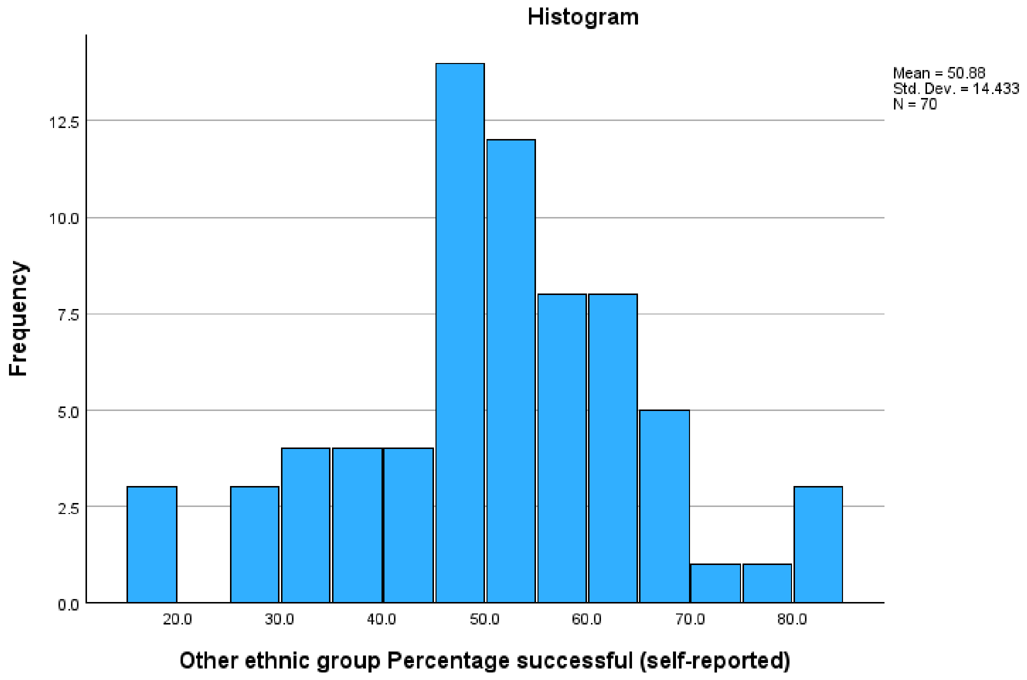

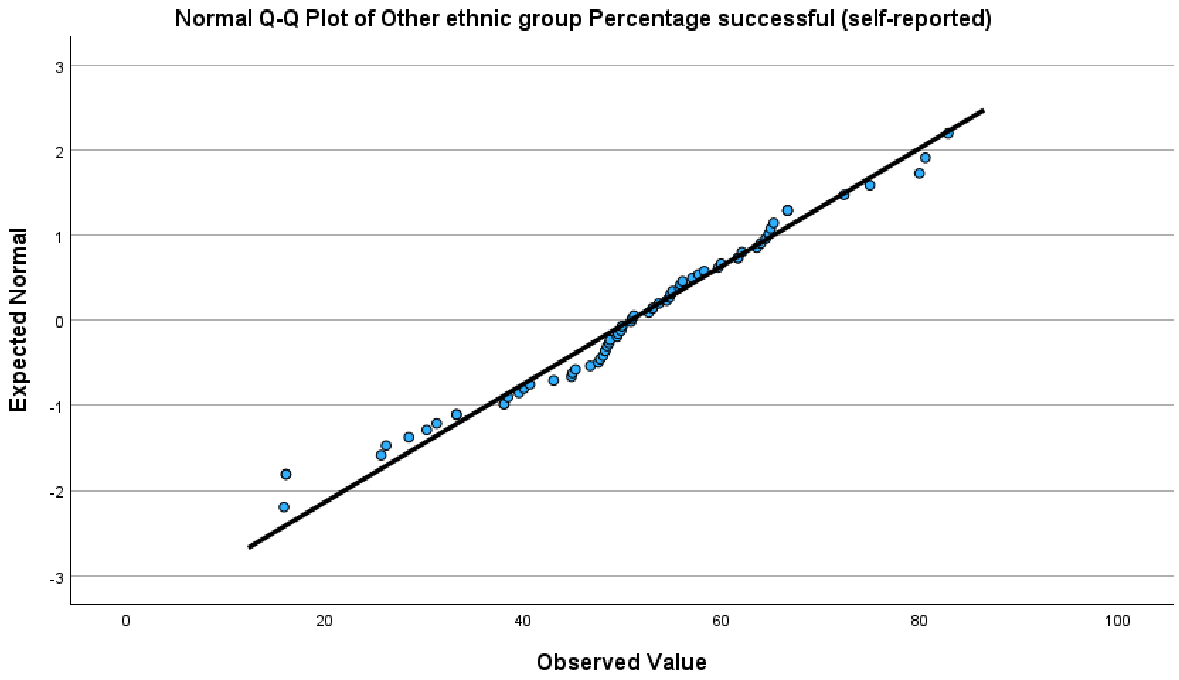

Not stated

Tests of Normality						
	Kolmogorov-Smirnov[a]			Shapiro-Wilk		
	Statistic	df	Sig.	Statistic	df	Sig.
Not stated Percentage successful (self-reported)	.064	117	.200*	.988	117	.379
a. Lilliefors Significance Correction						
*. This is a lower bound of the true significance.						

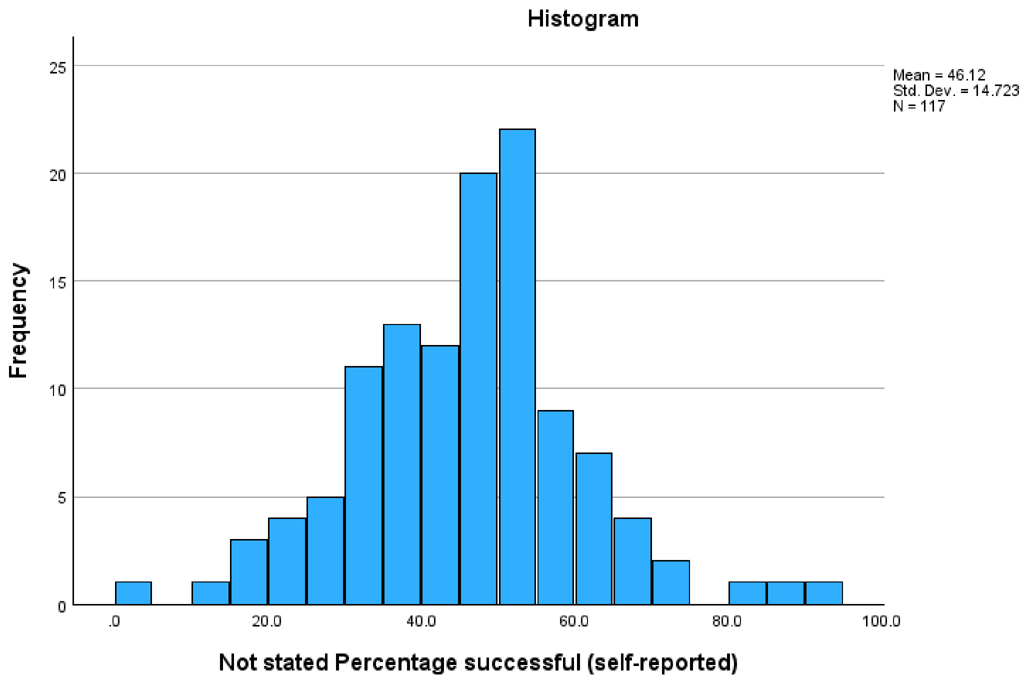

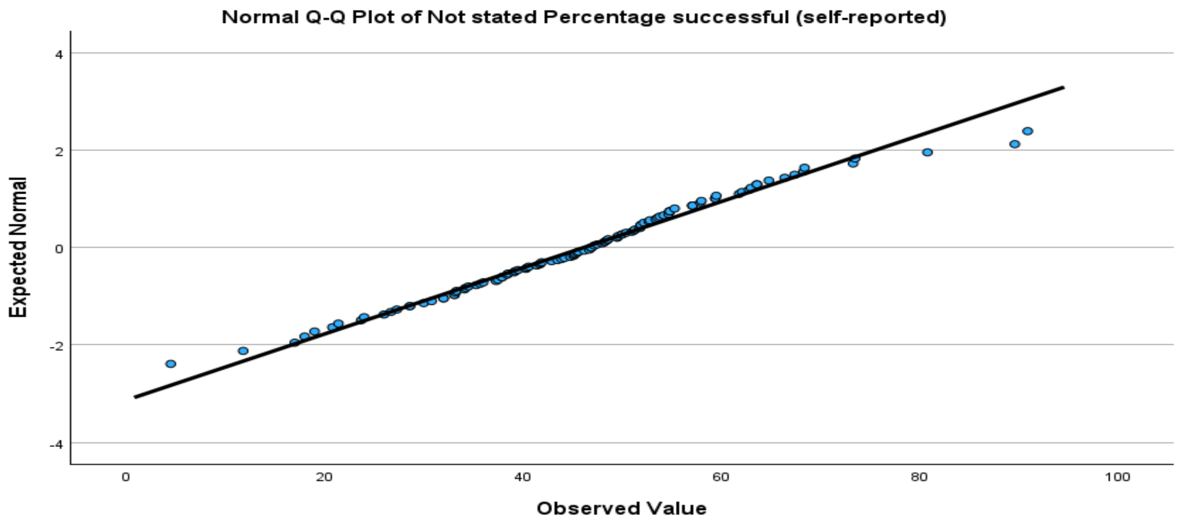

Diff_White_Asian (paired t-test)

Tests of Normality						
	Kolmogorov-Smirnov[a]			Shapiro-Wilk		
	Statistic	df	Sig.	Statistic	df	Sig.
Diff_White_Asian	.081	111	.070	.983	111	.185

a. Lilliefors Significance Correction

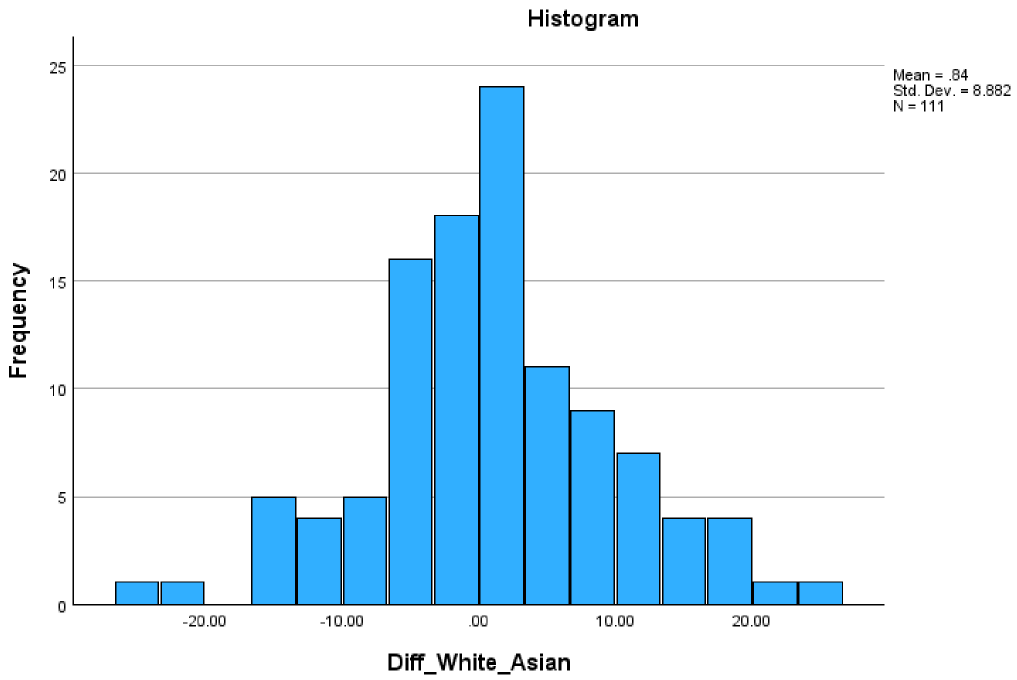
Histogram
Mean = .84
Std. Dev. = 8.882
N = 111
Frequency
0
5
10
15
20
25
-20.00
-10.00
.00
10.00
20.00
Diff_White_Asian

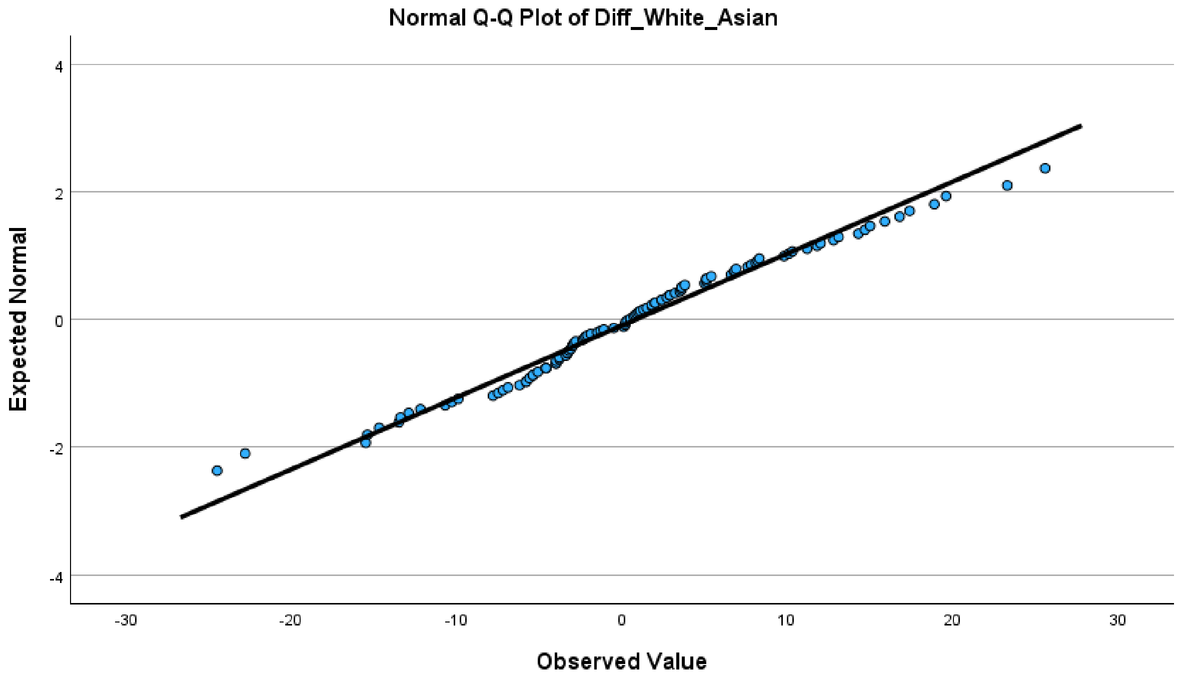
Normal Q-Q Plot of Diff_White_Asian
Expected Normal
4
2
0
-2
-4
-30
-20
-10
0
10
20
30
Observed Value
