# Supplementary material for: Ethnic and Regional Disparities in Self-Reported Smoking Cessation Success: A Cross-Sectional Analysis of Local Authority-Level Data in England
Source: Healthcare (Basel). 2026 Aug 25;14(17):2715. doi: 10.3390/healthcare14172715 (PMC13565527; doi:10.3390/healthcare14172715)
Supplement: Supplementary file 1 [file healthcare-14-02715-s001.zip › Supplementary File S2_ Assumption Diagnostics.pdf]

Supplementary File S2. Summary statistics for aggregated ethnic categories used in ANOVA analyses

Ethnic Category	n	Mean Quit Success (%)	SD (+/-)
White	162	52.5	9.2
Asian or Asian British	111	50.8	12.6
Black or Black British	72	51.6	11.6
Mixed	85	50.0	11.6
Other	70	50.9	14.4
Not Stated	117	46.1	14.7
