# Supplementary material for: Ethnic and Regional Disparities in Self-Reported Smoking Cessation Success: A Cross-Sectional Analysis of Local Authority-Level Data in England
Source: Healthcare (Basel). 2026 Aug 25;14(17):2715. doi: 10.3390/healthcare14172715 (PMC13565527; doi:10.3390/healthcare14172715)
Supplement: Supplementary file 1 [file healthcare-14-02715-s001.zip › Supplementary File S1_Consort flow diagram.pdf]

## Supplementary File S1: CONSORT-Style Flow Diagram for Analytic Samples

### Identification

- NHS Stop Smoking Services dataset includes:
    - 162 Local Authorities (LAs)
    - 9 regional summary rows
    - 1 England national summary row

### Screening

- Regional summary rows removed (not individual LAs)
- England national row removed (not an LA)

**Remaining: 162 Local Authorities**

### Eligibility

- Ethnicity-specific quit-success data extracted from Table 2.5
- NHS suppression rules remove LA-category cells with small or unstable quit-date counts (suppressed cells appear as * or z in the dataset)

**Remaining LA-category observations after suppression: 972** (used for correlation analysis)

### Inclusion for Kruskal–Wallis

- Rows missing quit-success percentage removed

**Remaining LA-category observations: 617** (used for Kruskal-Wallis test)

### Category-specific availability

(used for ANOVA and Mann–Whitney tests)

| Ethnic Category | n LAs contributing data |
|---|---:|
| **White** | 162 |
| **Asian or Asian British** | 111 |
| **Black or Black British** | 72 |
| **Mixed** | 85 |
| **Other** | 70 |
| **Not Stated** | 117 |
